# German Experience with a Novel Balloon-Expandable Heart Valve Prosthesis for Transcatheter Aortic Valve Implantation—Outcomes of the MYLAND (MYvaL germAN stuDy) Study

**DOI:** 10.3390/jcm13113163

**Published:** 2024-05-28

**Authors:** Timm Ubben, Eike Tigges, Won-Keun Kim, Andreas Holzamer, Ingo Breitenbach, Ralf Sodian, Jürgen Rothe, Willibald Hochholzer, Samer Hakmi, Franz-Josef Neumann

**Affiliations:** 1 Department of Cardiology, Asklepios Clinic St. Georg, 20099 Hamburg, Germany; e.tigges@asklepios.com; 2 Department of Cardiology and Cardiac Surgery, Kerckhoff Heart Center, 61231 Bad Nauheim, Germany; w.kim@kerckhoff-klinik.de; 3 Department of Cardiothoracic Surgery, University of Regensburg Heart Center, 93053 Regensburg, Germany; andreas.holzamer@klinik.uni-regensburg.de; 4 Department of Cardiothoracic Surgery and Vascular Surgery, Clinic of Braunschweig, 38120 Braunschweig, Germany; i.breitenbach@skbs.de; 5 Department of Cardiovascular Surgery, MediClin Herzzentrum Lahr/Baden, 77933 Lahr, Germany; ralf.sodian@mediclin.de; 6 Department of Cardiology and Angiology, Faculty of Medicine, Medical Center—University Heart Freiburg, University of Freiburg, 79189 Bad Krozingen, Germany; juergen.rothe@uniklinik-freiburg.de (J.R.); franz-josef.neumann@uniklinik-freiburg.de (F.-J.N.); 7 Department of Cardiology and Intensive Care Medicine, Klinikum Würzburg Mitte, 97070 Würzburg, Germany; 8 Department of Cardiothoracic Surgery, Asklepios Clinic St. Georg, 20099 Hamburg, Germany; s.hakmi@asklepios.com

**Keywords:** transcatheter aortic valve replacement, aortic stenosis, self-expanding THV

## Abstract

**Background:** The primary objective of this study was to evaluate the initial experience in Germany with the Meril Myval™ (MM) transcatheter heart valve (THV) system for the treatment of severe symptomatic aortic valve stenosis. The MM THV is a novel balloon-expandable valve with an expanded sizing matrix. Contemporary patients undergoing TAVI with the established Edwards Sapien™ (ES) THV served as the comparator group. **Methods**: Between 1st March and 31 August 2020 a total of 134 patients (33% female, 80.1 ± 6.7 years; EuroScore II 4.7 ± 4.8) underwent TAVI with an MM (95% transfemoral) for severe aortic stenosis at six German tertiary care centers. **Results:** Correct positioning of the THV was achieved in 98.5% (n = 132). Mean aortic gradients (MPG) were reduced from 42 ± 14 mmHg to 11 ± 5 mmHg. Mild postprocedural paravalvular leak (PVL) was observed in 62% (n = 82) patients, whereas only one patient had more than mild PVL. New permanent pacemaker implantation (PPI) was indicated in 15 patients (11%). Major vascular complications occurred in 6.7% (n = 9) patients. The in-hospital combined incidence of all-cause death and stroke was 4.5% (n = 6). In the comparator group that included 268 patients, the 30-day incidences of PPI, major vascular complications, and the composite of all-cause death and stroke were 16%, 1.9%, and 7.1%, respectively; MPGs were reduced from 44 ± 15 mmHg to 12.8 ± 4.6 mmHg and the more than mild PVL occurred in 0.7%. **Conclusions:** The MM is a promising novel THV system, with performance comparable to the established ES THVs. These findings await confirmation by ongoing randomized trials.

## 1. Introduction

**Figure 1:**
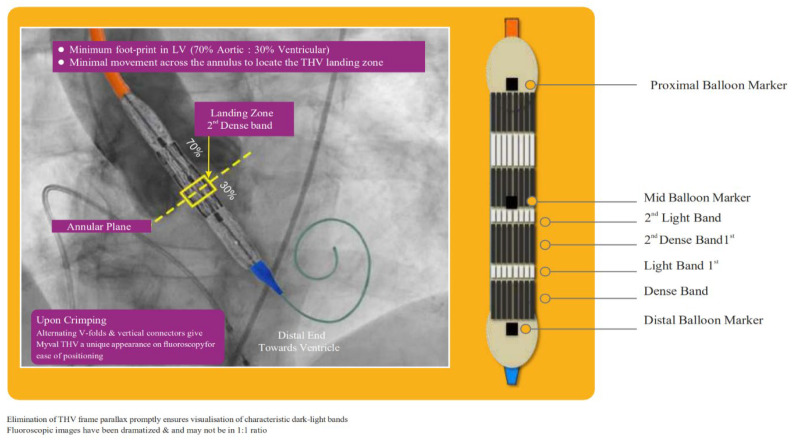
Meril Myval THV and visualization of the relationship between landing zone and annular plane.

**Figure 2:**
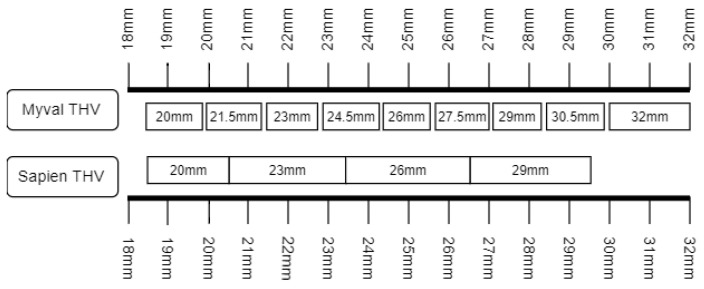
Sizing scheme for Meril Myval and Edwards Sapien THV system.

The Meril Myval™ (MM) balloon-expandable transcatheter heart valve (THV) system (Meril Life Sciences Pvt. Ltd., Vapi, India) was introduced towards the end of 2018. Until then the Edwards Sapien (ES) (Edwards Lifesciences, Irvine, CA, USA) was the only balloon-expandable THV available. While TAVI has evolved as a standard procedure for patients with AS [1,2,3], the need for new permanent pacemaker implantation (PPI), valve embolism, paravalvular leakage (PVL), annular rupture, and second transcatheter heart valve implantation after TAVI still remain limitations [1,2,3]. Higher grade paravalvular leakage (PVL) and significant conduction disturbances leading to new PPI following TAVI procedure affect morbidity and mortality following TAVI procedures [3,4,5]. These events are observed more frequently in borderline annulus size with limited options for appropriate planning measures [6]. The use of ES compared to self-expanding THV systems was associated with higher rates of annular rupture [7]. This necessitates the need for additional sizes in order to minimize the extent of suboptimal sizing [6,7,8]. The novel Myval™ THV system (Meril Life Sciences Pvt. Ltd., India) was designed with an expanded size matrix including intermediate (21.5, 24.5, and 27.5 mm) and extra-large (30.5 and 32 mm) sizes (Figure 1 and Figure 2) [8]. Moreover, the current iteration of the MM system, Octacor™, has dedicated design features that facilitate commissural alignment.

The current study is a retrospective analysis of the acute safety and efficacy of the MM THV system in the first German experience with this THV. Contemporary ES implantations served as a reference.

## 2. Methods

### 2.1. Study Design

The MYLAND study is an investigator-initiated, multicenter, retrospective, observational study to evaluate the initial experience in Germany with the MM THV system. The study involves a retrospective collection of data without any formal hypothesis testing. The study was conducted at six high-volume TAVI centers. The comparator group consisted of contemporary patients treated with an ES THV system.

Following contemporary European guidelines, our multidisciplinary heart team assessed patients for TAVI to determine eligibility, procedure feasibility, access route, valve type, and size. The pre-TAVI assessment included transthoracic and, if necessary, transesophageal echocardiography, as well as coronary angiography and computed tomography angiography. Systolic annular dimensions were obtained from planimetric area CTA measurements, and the effective annulus diameter as well as the annulus perimeter were calculated. TAVI was performed under general anesthesia or conscious sedation as deemed appropriate by the heart team. Post-interventional antithrombotic treatment typically consisted of dual-antiplatelet therapy with acetylsalicylic acid (100 mg/day) and clopidogrel (75 mg/day) for 6 months, followed by lifelong acetylsalicylic acid. Generally, patients who require oral anticoagulation were not given antiplatelet therapy at the same time.

Patients were monitored for at least 24 h after TAVI. The indication for permanent pacemaker implantation was based on individual shared decision-making between the patient and the attending medical team at each study center. In general, a new pacemaker was implanted for (a) intermittent or persistent pacemaker dependency during the surveillance period on temporary pacemaker, (b) intermittent high-grade atrioventricular (AV) block, (c) new left bundle branch block with AV block I, (d) new bifascicular block, (e) left bundle branch block with increasing QRS width or increasing AV block I.

### 2.2. Study Population

Included in this study were patients who underwent TAVI for severe symptomatic aortic valve stenosis with either MM THV or ES 3 or ES Ultra THV at the study centers between 1 March 2020 and 31 August 2020. Exclusion criteria were pre-existing prosthetic heart valves in aortic position and patients who underwent an emergent TAVI or TAVI with mechanical circulatory support. Contributing centers were the University Hospital Regensburg, Städtisches Klinikum Braunschweig, MediClin Heart Center Lahr, University Heart Centre Freiburg, Bad Krozingen, Kerckhoff Klinik Bad Nauheim and Asklepios Klinik St. Georg in Hamburg.

During the study period, all consecutive patients treated in these centers with an MM THV were included. For the comparator group, consecutive patients treated with an ES THV were chosen until twice the number of patients treated with an MM at each center was reached.

### 2.3. Outcomes

The aim of the study was to analyze in-hospital outcomes. Outcomes were assessed by each center according to the Valve Academic Research Consortium 3 criteria [9].

### 2.4. Statistical Analysis

The statistical report comprises the analysis of the MYLAND study with summary tables of all data, pre- and post-intervention analysis of mean AV gradient, and estimates of mortality and morbidity events. Discrete variables are presented as percentages (counts) and compared using the chi-squared test. Continuous variables were reported as mean ± standard deviation or median (interquartile range) and compared using the Student’s *t*-test or the Mann–Whitney U test, respectively. Mean differences (differences in proportions) are shown between the groups in case of baseline/anamnestic data. Nominally significant results can be derived from confidence intervals. As the intervals do not include 0, *p*-values are below 5%. The statistical analysis was performed using R Core Team 2021. The statistical analyses and interpretation of the data were authorized by all authors. The authors attest to the accuracy of the data and of all analyses. The ethics committee of the General Medical Council for the city of Hamburg approved the study and waived the need for informed consent.

## 3. Results

### 3.1. Baseline Characteristics

During the study period, 134 patients were treated with the MM THV via femoral access in 95% (n = 127) and via alternative access sites in 5.2% (n = 7) of the cases (transcarotid in 0.7% (n = 1), transaxillary in 3.7% (n = 5), transapical in 0.7% (n = 1). The mean age was 81 ± 5.8 years and 32% (n = 45) were female. The comorbidities cumulated in a median EuroScore II of 3.4 (2.1, 5.1) and STS-Score of 3.2 (2.0, 4.8). Additional baseline demographic and clinical characteristics are provided in Table 1.

The computed tomography (CT) parameters and hemodynamic data are shown in Table 1. All patients had severe AS with a mean gradient of 42 ± 14 mmHg and a mean EOA of 0.74 ± 0.17 cm^2^. CT showed tricuspid anatomy of the aortic valve in 89% (n = 119), bicuspid anatomy in 9.8% (n = 13), whereas cusp anatomy could not be determined in 0.8% (n = 1). The mean AV calcium score was 3573 ± 3147 AU, the mean area-derived AV annulus diameter was 25.44 ± 2.27 mm, and the mean AV annulus area was 497 ± 102 mm^2^. The maximum annulus area treated was 755 mm^2^ and the minimal annulus area was 320 mm^2^.

The comparator group comprised 268 consecutive patients treated with an ES THV, among them 120 with an ES 3 TVH and 148 with an ES Ultra via femoral access in 94.8% (n = 253). Alternative access sites in 5.2% (n = 14) were transcarotid in 0.3 (n = 1), transaxillary in 2.9% (n = 8), and transapical 1.8% (n = 5) cases. As shown in Table 1, baseline demographic and clinical characteristics as well as CT parameters and hemodynamic data in the comparator group were similar to those in the study group.

### 3.2. Procedural Outcomes

**Figure 3:**
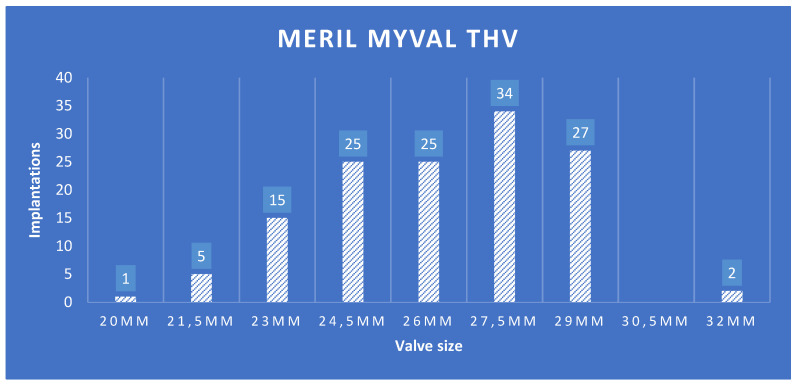
Distribution of implanted prosthesis sizes for the Meril Myval THV.

**Figure 4:**
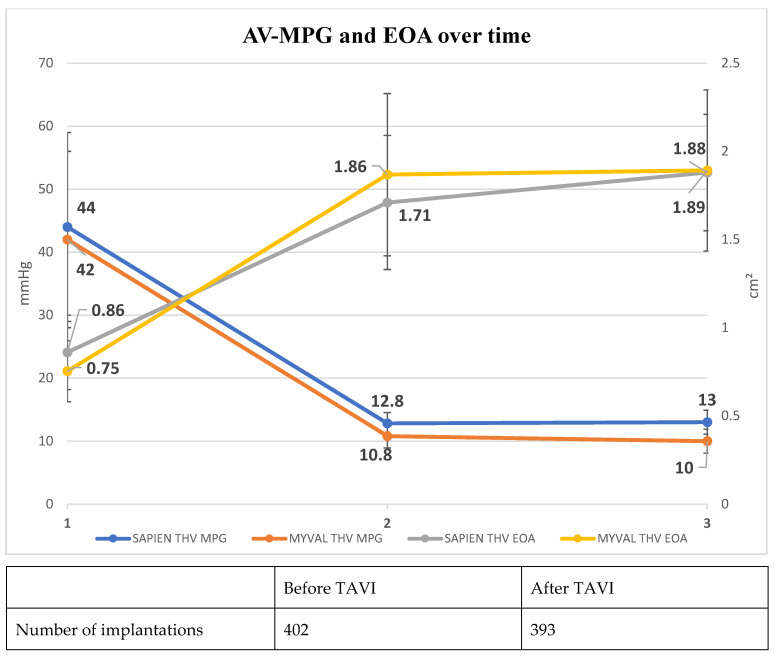
Echocardiographic aortic valve mean gradient (AV-MPG) and effective orifice area (EOA) before, after TAVI, and during follow-up.

The distribution of valve sizes in the MM group is shown in Figure 3. Correct positioning of the THV was achieved in 98.5% (n = 132), technical success was 91.8% with failures due to vascular complications in nine (6.7%) patients, valve embolization in one patient (0.7%) and death before successful positioning in one patient (0.7%). As shown in Figure 4, the mean aortic valve gradient significantly decreased to 10.8 ± 4.5 mmHg, and the mean EOA increased to 1.87 ± 0.46 cm^2^. The maximum aortic valve gradient was reduced from 69 ± 22 mmHg to 19 ± 7 mmHg. No or trace postprocedural paravalvular leak (PVL) was observed in 82 (62%) patients, whereas only 1 patient had more than mild PVL.

**Figure 5:**
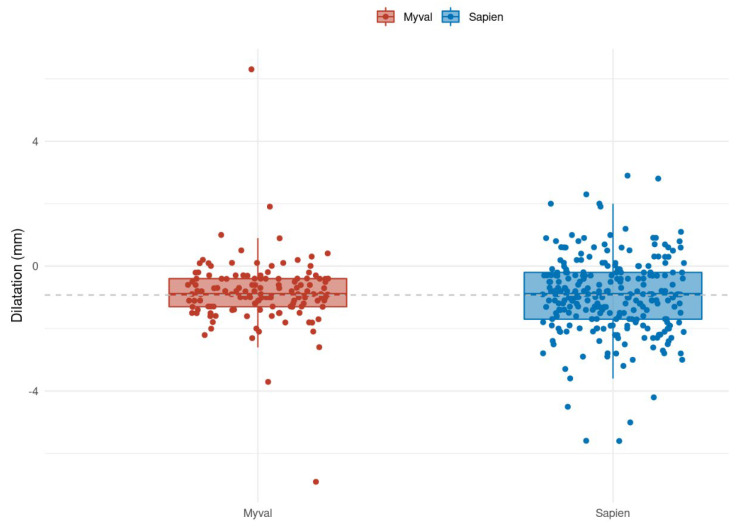
Boxplot and jitter plot of the difference between the area-derived annulus diameter and the chosen prosthesis size. Dashed line represents mean difference across groups.

The estimated difference between annular diameter and valve size was −0.85 mm ± 1.09 mm, as shown in Figure 5.

**Figure 6:**
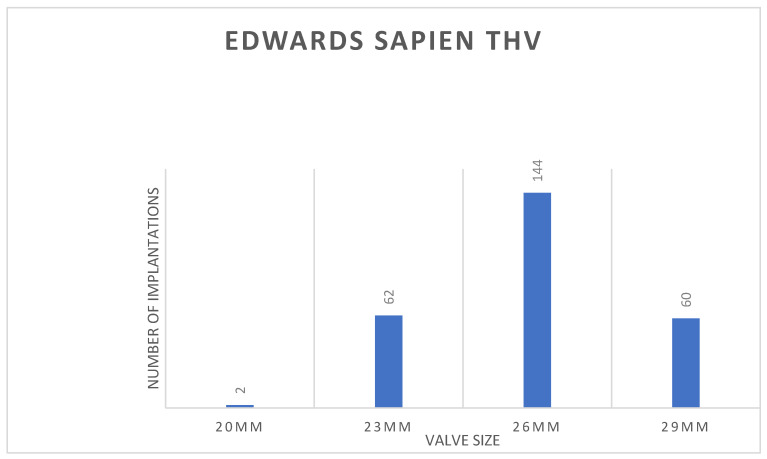
Distribution of implanted prosthesis sizes for the Edwards Sapien THV.

With the distribution of the ES sizes shown in Figure 6, technical success in the comparator group was 97%, with one valve embolization and one annular rupture. As shown in Figure 4, reductions in aortic valve gradients and increases in EOA were almost identical to those in the study group. Only one patient showed PVL more than mild (0.7%), while 62% (n = 96) had no or trace AR.

The estimated difference between annular diameter and valve size with ES implantation (0.97 mm ± 1.22) was numerically higher after ES implantation than after MM implantation (Figure 5).

### 3.3. Clinical Outcomes

Clinical outcomes are summarized in Table 2. With the MM TVH, major vascular complications occurred in 6.7% (n = 9), while minor vascular complications were observed in 5.2% (n = 7). Life-threatening bleedings occurred in 3.7% (n = 5). New PPI was performed in 11% (n = 15). In-hospital all-cause mortality was 2.2% (n = 3) and cardiac mortality was 1.5% (n = 2). Three patients (2.2%) suffered a stroke. The median post-procedural hospital stay was 7 days (IQR 5, 9).

As shown in Table 2, clinical outcomes after ES implantation were similar to those after MM implantation. At 1.9% (n = 5), only the incidence of major vascular complications was lower after ES than after MM, with nominal statistical significance.

## 4. Discussion

Our study shows the initial experience with the new MM THV system in the treatment of patients with severe AS. The MM THV was highly efficacious in reducing mean aortic gradients to roughly 10 mmHg on average with a robust increase in EOA and 99.3% had none to mild PVL. These performance markers were well in line with the ES THV benchmark [10,11]. This was shown by comparison with our contemporary group of patients with ES implantations as well as with historical data published in the literature. New PPI was performed in 11% of the patients after MM implantation. This rate was somewhat higher than the rates reported in previous studies on ES TVHs such as PARTNER-3 [10], but numerically lower than the new PPI rate in our comparator group [11]. The overall high rates of new PPI may be a consequence of a more liberal indication for PPI compared to international practice. The risk of the two most dreaded complications of TAVI, death and stroke, was low after MM implantation and again in the same range as in the comparator group. This was also true for other complications of TAVI shown in Table 2, except for major vascular complications.

Major vascular complications occurred in 6.9% of the patients with an MM THV, which was higher than expected, whereas the risk of major vascular complications in the ES group was within the expected range at 1.9%. Three aspects deserve consideration in this respect. First, a play of chance cannot be excluded, as the nominally significant *p*-value is not conclusive because of multiple testing. Second, limited experience with the specific design of the dedicated MM sheath may have contributed to the higher-than-expected incidence of vascular problems. Finally, the MM THV has a somewhat larger vascular access than the ES THV, because the MM THV is fully crimped onto the balloon before insertion. Thus, although a 14Fr sheath is used for all MM sizes, there is a need to predilate the 14Fr sheath with an 18Fr dilatator before the insertion of larger valve sizes. With the ES, however, 20 mm and 26 mm valve sizes are fully 14Fr compatible and only the 29 mm valve requires a 16Fr sheath.

Sacrificing some of the device slenderness for the sake of a fully crimped balloon prosthesis assembly prior to insertion may, however, offer some advantages apart from streamlining deployment. Unlike the ES TVH, it allows the device to be withdrawn after insertion, a feature that fortunately was not needed in the current series. Moreover, precrimping avoids potential risks of the loading maneuver in the aorta, which may be particularly relevant in cases with heavily calcified or extreme kinking in the aorta or with alternative access sites, such as subclavian, transaxillary, or transcarotid access. The size of the current study, however, was not sufficient to address the impact of these potential advantages.

Compared to the ES THV the MM THV features a wider range of available valve sizes (20 mm–32 mm) and also offers a more granular sizing matrix with the presence of intermediate sizes (e.g., 21.5 mm, 24.5 mm, 27.5 mm, 30.5 mm). There was a widespread use of intermediate sizes (47% [n = 64] of the MM THV patients were treated with intermediate sizes) in the MM THV group. The availability of intermediate and extra-large sizes allows the MM THV to differ by 1.5 mm (20, 21.5, 23, 24.5, 26, 27.5, 29, 30.5, and 32 mm) as opposed to the difference of 3 mm between two sizes of ES THV (20, 23, 26, and 29 mm). The expanded size matrix of MM THV allows appropriate sizing without the need for excessive over- or under-expansion. The operators, therefore, could use the correct sizing of the THV when using MM with the aim of achieving optimal outcomes. This is further corroborated in an operator-based survey where 42% of implants were conducted using the intermediate sizes of MM [12]. Also, a wider range of annulus sizes could be treated with a maximum annulus area in the MM THV group of 755 mm^2^ and of 688 mm^2^ in the ES THV group. The latter already transgressed the approved upper limit for ES of 680 mm^2^ in seven patients. It has been hypothesized that the extended and more granular sizing matrix of the MM compared with the ES TVH would improve the match between prosthesis size and annulus size and thereby minimize the risks of annulus rupture, PVL, and new PPI. We were unable to prove this hypothesis in the current initial experience with the MM THV. Nevertheless, some numerical differences pointed in this direction: The estimated difference between annular diameter and valve size with ES implantation and the new PPI rate were at least numerically higher after ES implantation than after MM implantation and the only annulus rupture occurred in the ES group.

## 5. Conclusions

Our study shows that the performance of the new MM THV is similar to the established ES THV. Our initial experience with the MM THV reported here has helped break the ground for randomized trials comparing the MM THV to established valves of the SAPIEN^TM^ or EVOLUTE^TM^ family. Unlike our study, these studies will be able to address the potential benefits of the extended and more granular sizing matrix. Particular attention should be paid to vascular access management to improve outcomes beyond our experience.

## 6. Limitations

This was a retrospective study conducted at six centers in Germany reflecting the initial experience with a new THV type. Thus, the influence of a learning curve as well as bias towards uncomplicated cases cannot be excluded. For these reasons, we refrain from formal hypothesis testing. The sample size is limited and, hence, the study is unable to address rare events.

## Figures and Tables

**Table 1:** Baseline characteristics.

Characteristics	Meril Myval, n = 134	Edwards Sapien, n = 268	Difference (95% CI)	*p*-Value
Gender (female)	34 (45)	32 (86)	1.5 (−8.8; 12)	0.76
Mean age (years)	81.0 (5.9)	79.7 (7.0)	1.3 (−0.04; 2.6)	0.072
Mean body mass index (kg/m^2^)	26.9 (4.2)	27.3 (5.0)	−0.43 (−1.4; 0.5)	0.69
Hypertension	92 (123)	93 (249)	1.1 (−7.3; 5.0)	0.68
Diabetes mellitus	25 (34)	31 (84)	−6.0 (−16; 3.8)	0.21
Prior coronary artery graft bypass	14 (19)	11 (29)	3.4 (−4.2; 11)	0.32
Prior percutaneous coronary intervention	44 (59)	43 (114)	1.5 (−9.3; 12)	0.77
Prior stroke	8.2 (11)	8.6 (23)	−0.37 (−6.5; 5.7)	0.89
Chronic renal disease	23 (32)	24 (63)	0.37 (−8.8; 9.6)	0.31
Mean creatinine (mg/dL)	1.14 (0.69)	1.36 (1.26)	−0.22 (−0.41; −0.2)	0.066
Existing PPI	13 (18)	13 (34)	0.8 (0.1; 1–7)	0.17
log. EuroScore (%)	16 (12)	18 (16)	−2.3 (−5.2; 0.66)	0.16
EuroSCORE II (%)	4.5 (3.4)	4.9 (5.4)	−0.42 (−1.3; 0.48)	0.43
STS-Score (%)	4.7 (6.1)	3.9 (3.7)	0.82 (−0.56; 2.2)	0.17
Echocardiography				
Mean aortic valve gradient (mmHg)	42 (14)	44 (15)	1.21 (−1.79; 4.21)	0.38
Max aortic valve gradient (mmHg)	69 (22)	71 (24)	1.6 (−3.25; 6.46)	0.52
Effective valve orifice area (cm^2^)	0.74 (0.17)	0.86 (2.11)	0.12 (−0.13; 0.37)	0.51
**Valve anatomy**				
Tricuspid valve anatomy	89 (119)	88 (237)	−0.01 (−0.07; 0.05)	0.73
Bicuspid valve anatomy	9.8 (13)	11 (29)	0.01 (−0.05; 0.07)	0.74
Mean calcium score (AU)	3573 (3147)	3627 (2206)	54.39 (−766; 810)	0.88
Mean area-derived diameter (mm)	25.44 (2.27)	24.99 (2.22)	−0.27 (−0.07; 0.05)	0.43
Annulus area (mm^2^)	497 (102)	482 (76)	−14 (−36,7; 7.88)	0.18
LVOT diameter (mm)	25.43 (2.76)	24.91 (3.90)	−52 (−1.27; 0.23)	0.23

**Table 2:** Clinical outcomes at discharge.

Characteristics	Meril Myval, n = 134	Edwards Sapien, n = 268	*p*-Value
**All-cause mortality**	2.2 (3)	3.4 (9)	0.76
Cardiac mortality	1.5 (2)	2.3 (6)	0.72
Non-cardiac mortality	0.7 (1)	1.1 (3)	>0.99
**Stroke**	2.2 (3)	1.5 (4)	0.69
Disabling stroke	0.7 (1)	0.8 (2)	>0.99
Non-disabling stroke	1.5 (2)	0.8 (2)	0.60
**New pacemaker implantation**	11 (15)	16 (42)	0.23
**Annular rupture**	0 (0)	0.4 (1)	>0.99
**New onset atrial fibrillation**	3.7 (5)	1.5 (4)	0.17
**Cerebrovascular events**	1.5 (2)	1.5 (3)	>0.99
**Acute renal failure**	2.2 (3)	4.5 (12)	0.403
**Type 3 bleeding**	3.7 (5)	2.7 (7)	0.55
**Endocarditis**	0 (0)	0 (0)	>0.99
**Myocardial infarction**	0 (0)	0 (0)	>0.99
**Major vascular complication**	6.7 (9)	1.9 (5)	0.02
**Minor vascular complication**	5.2 (7)	2.7 (7)	0.25

## Data Availability

Data are contained within the article.

## Abbreviations

AR	aortic regurgitation
AS	aortic stenosis
AV	aortic valve
BE	ballon-expandable
CT	computed tomography
EOA	effective orifice area
ES	Edwards Sapien
Fr	French
MM	Meril Myval
MPG	mean pressure gradient
PPI	permanent pacemaker implantation
PVL	paravalvular leakage
TAVI	transcatheter aortic valve implantation
THV	transcatheter heart valve
